# High-Fat Diet-Induced Obesity Causes Sex-Specific Deficits in Adult Hippocampal Neurogenesis in Mice

**DOI:** 10.1523/ENEURO.0391-19.2019

**Published:** 2020-01-02

**Authors:** Lisa S. Robison, Nathan M. Albert, Lauren A. Camargo, Brian M. Anderson, Abigail E. Salinero, David A. Riccio, Charly Abi-Ghanem, Olivia J. Gannon, Kristen L. Zuloaga

**Affiliations:** Department of Neuroscience and Experimental Therapeutics, Albany Medical College, Albany, New York 12208

**Keywords:** hippocampus, metabolic disorders, microglia, neurogenesis, prediabetes, sex differences

## Abstract

Adult hippocampal neurogenesis (AHN) is suppressed by high-fat (HF) diet and metabolic disease, including obesity and type 2 diabetes. Deficits in AHN may contribute to cognitive decline and increased risk of dementia and mood disorders, which have higher prevalence in women. However, sex differences in the effects of HF diet/metabolic disease on AHN have yet to be thoroughly investigated. Herein, male and female C57BL/6J mice were fed an HF or control (CON) diet from ∼2 to 6 months of age. After 3 months on the diet, mice were injected with 5-ethynyl-2′-deoxyuridine (EdU) then killed 4 weeks later. Cell proliferation, differentiation/maturation, and survival of new neurons in the dentate gyrus were assessed with immunofluorescence for EdU, Ki67, doublecortin (DCX), and NeuN. CON females had more proliferating cells (Ki67^+^) and neuroblasts/immature neurons (DCX^+^) compared with CON males; however, HF diet reduced these cells in females to the levels of males. Diet did not affect neurogenesis in males. Further, the numbers of proliferating cells and immature neurons were inversely correlated with both weight gain and glucose intolerance in females only. These effects were robust in the dorsal hippocampus, which supports cognitive processes. Assessment of microglia in the dentate gyrus using immunofluorescence for Iba1 and CD68 uncovered sex-specific effects of diet, which may contribute to observed differences in neurogenesis. These findings demonstrate sex-specific effects of HF diet/metabolic disease on AHN, and highlight the potential for targeting neurogenic deficits to treat cognitive decline and reduce the risk of dementia associated with these conditions, particularly in females.

## Significance Statement

Poor diet and metabolic disease, including obesity and type 2 diabetes, are associated with an increased risk of neurodegenerative and neuropsychiatric disorders, including Alzheimer’s disease, anxiety, and depression. Impaired adult hippocampal neurogenesis may be one mechanism linking these conditions; however, it is unknown whether there are sex-specific effects of high-fat diet/metabolic disease on neurogenesis, which could underlie the observed sex difference in these conditions (females more adversely affected than males). We report that high-fat diet/metabolic disease impairs cell proliferation and the number of neuroblasts/immature neurons in the dorsal hippocampus, a region that supports cognitive function, in females only. Sex-specific effects of diet on microglia in the subgranular zone were also apparent.

## Introduction

Neurogenesis involves the proliferation, migration, differentiation, survival, and integration of new neurons into existing circuitry, playing a role in brain plasticity (Christie and Cameron, 2006). One region of the brain in which neurogenesis persists through adulthood in many species, including humans and rodents, is the subgranular zone (SGZ) of the dentate gyrus within the hippocampus (Kempermann et al., 2015). This region can be split along its longitudinal axis into the dorsal hippocampus (septal pole), which is involved in cognitive processes, and the ventral hippocampus (temporal pole), which is involved in emotion processing; these are analogous to the posterior and anterior portions of the hippocampus, respectively, in primates (Fanselow and Dong, 2010). Therefore, adult hippocampal neurogenesis (AHN) plays a role in both cognitive functions, such as learning and memory, and affective processes, including mood regulation and reactivity to stress (Tanti and Belzung, 2013).

Hippocampal atrophy and dysregulated AHN are seen in the brains of patients with neurodegenerative and neuropsychiatric illnesses, including Alzheimer’s disease (AD), anxiety, and depression, while animal models of these diseases also exhibit altered neurogenesis (Wang et al., 2003; Cole et al., 2011; Hill et al., 2015; Miller and Hen, 2015; Chan et al., 2016; Unger et al., 2016; Moreno-Jiménez et al., 2019). Impaired AHN is hypothesized to contribute to symptoms of these conditions, as interventions that promote neurogenesis alleviate symptoms, and medications used to treat these disorders enhance AHN (Santarelli et al., 2003; Jin et al., 2006; Boldrini et al., 2009; Blanchard et al., 2010; Valero et al., 2011; Hill et al., 2015; Richetin et al., 2015; Tapia-Rojas et al., 2016). Therefore, factors that affect AHN also likely influence risk and severity of these diseases. In addition to pharmacological interventions, AHN is altered by environmental factors, including physical activity, stress, and learning (Tanapat et al., 1998; Gould et al., 1999; van Praag et al., 1999). Interestingly, some factors not only influence specific stages of neurogenesis (e.g., proliferation or new neuron survival), but also affect neurogenesis in a subregion- and/or sex-specific manner (Westenbroek et al., 2004; Tanti et al., 2012; Tanti et al., 2013; Yagi and Galea, 2019). This may contribute to sex differences in the observed prevalence, severity, and rate of cognitive decline seen in dementia and depression, with females being more adversely affected (McPherson et al., 1999; Gutiérrez-Lobos et al., 2002; Irvine et al., 2012; Hebert et al., 2013; Beam et al., 2018; Buckley et al., 2019; Eid et al., 2019).

High-fat (HF) diet and metabolic disease, such as obesity and type 2 diabetes, are associated with dysregulated neurogenesis (Park et al., 2010; Ho et al., 2013; Nam et al., 2016; Nam et al., 2017; Tanokashira et al., 2018; Tang et al., 2019). Poor diet and metabolic disease are also associated with cognitive impairment, increased risk/severity of Alzheimer’s disease, and anxiety and depression (Jacka et al., 2010; Xu et al., 2011; Boitard et al., 2012; Spauwen et al., 2013; Boitard et al., 2014; Knight et al., 2014; Chatterjee et al., 2016; Klein et al., 2016; Zuloaga et al., 2016; Shalev and Arbuckle, 2017). Specifically, overweight/obesity is associated with an increased risk ratio of 1.41 for dementia and odds ratio of 1.27–1.55 for depression (Luppino et al., 2010; Pedditzi et al., 2016). Deficits in neurogenesis may be one mechanism linking poor diet and metabolic disease to an increased risk for dementia and mood disorders (Ho et al., 2013). There is evidence to suggest that metabolically unhealthy women are at greater risk for dementia than men (Chatterjee et al., 2016; Gannon et al., 2019). Additionally, there is a sex-specific association between depression and diabetes, with a significant relationship seen in women only (Demmer et al., 2015). However, little is known in regard to possible sex differences in the effects of HF diet and metabolic disease on neurogenesis that may be mediating these relationships. To our knowledge, no studies to date have determined the effects of HF diet/metabolic disease on the various stages of neurogenesis in a sex- and region-specific manner. Herein, we sought to do so using male and female C57BL/6J mice administered an HF diet from ∼2 to 6 months of age, which was previously shown to result in a similar prediabetic phenotype (weight gain and glucose intolerance) in both sexes (Salinero et al., 2018).

## Materials and Methods

### Animals and experimental design

**Figure 1.**
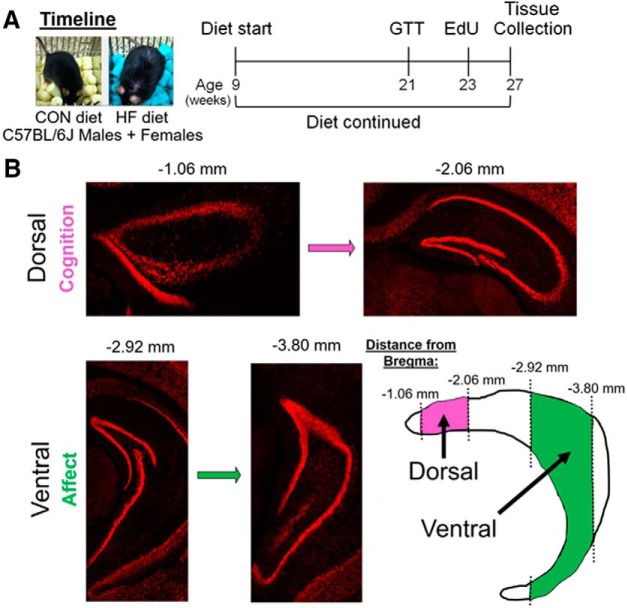
***A***, Timeline of the experiment. Male and female C57BL/6J mice were placed on a CON diet (10% fat) or an HF diet (60% fat) at 9 weeks of age, which was continued for the duration of the experiment. After ∼3 months on the diet, mice were subjected to GTT, then injected with EdU (3×) 2 weeks later. Four weeks after EdU injections, tissue collection was performed. ***B***, Breakdown of the hippocampus into dorsal (approximately −1.06 to approximately −2.06 mm from bregma) and ventral (approximately −2.92 to approximately −3.80 mm from bregma) subregions, which regulate cognitive and affective processes, respectively. Adapted from Tanti and Belzung (2013).

Male and female C57BL/6J mice were purchased from The Jackson Laboratory at ∼8 weeks of age. Mice were group housed (five per cage) at 70–72°F and 30–70% humidity, with a 12 h light/dark cycle (7:00 A.M. on/7:00 P.M. off). Following 1 week of acclimation on Purina Lab Diet 5P76, mice were split into equal groups by sex and placed on their respective treatment diets (*n* = 10/group). Mice received either an HF diet (60% fat from lard; 5.24 kcal/g; D12492, Research Diets) or a control (CON) diet (10% fat; 3.85 kcal/g; D12450B, Research Diets) and remained on the diet for the remainder of the study (18 weeks). Water was provided *ad libitum* throughout the entire experiment, and body weight was measured monthly throughout the dietary intervention period. At the end of the study, mice were ∼6 months of age. Following 12 weeks on the diet, mice underwent a glucose tolerance test (GTT), and allowed to recover for 2 weeks before 5-ethynyl-2′-deoxyuridine (EdU) injections (5 μl/g body weight, i.p.; 3×/d, 2 h apart). Four weeks after EdU injections, mice were deeply anesthetized with pentobarbital and transcardially perfused with 0.9% saline. Brains were rapidly removed and postfixed overnight in 4% formalin, then cryoprotected in 30% sucrose for at least 72 h, embedded in optimal cutting temperature compound, and stored at −80°C until cryosectioning. A timeline of the experiment can be seen in Figure 1*A*. All animal procedures were performed in accordance with the regulations of the animal care committee at Albany Medical College.

### GTT

At 12 weeks post-dietary intervention, mice were fasted overnight (16 h), and baseline blood glucose levels in saphenous vein blood were measured by glucometer (OneTouch Verio IQ). Each mouse received an intraperitoneal injection of 2 g/kg glucose (0.2 g/ml in Millipore water, sterile filtered), and blood glucose levels were remeasured at 15, 30, 60, 90, and 120 min post-glucose injection.

### Immunohistochemistry

Brains were sectioned in the coronal plane into six series of 40-μm-thick sections. Hippocampal sections (ranging from approximately −1.06 to approximately −3.80 mm from bregma) from one of these series were used for each panel of immunohistochemistry (IHC), resulting in ∼11 sections analyzed throughout the entirety of the hippocampus. Sections were washed with PBS with 0.01% sodium azide, permeabilized in 0.3% Triton X-100 in PBS (TPBS) with sodium azide for 1 h at room temperature, blocked in 4% donkey serum in 0.3% TPBS with sodium azide for 1 h at room temperature, and incubated at 4°C overnight with primary antibodies. For the analysis of neurogenesis, the primary antibodies used included rabbit anti-NeuN (neuronal-specific nuclear protein; 1:1000; ABN78, lot #3041797, Millipore), guinea pig anti-doublecortin (1:1000; AB2253, lot #3092504, Millipore), and rabbit anti-Ki67 (1:200; AB15580, lot #GR3197370-1, Abcam). For evaluation of the activity of microglia/macrophages, the primary antibodies used included goat anti-Iba-1 (1:1000; PA5-18039, lot #TI2638761, Thermo Fisher Scientific), and rat anti-CD68 (1:1000; MCA1957, lot #1708, Bio-Rad). Fluorescent secondary antibodies (Jackson ImmunoResearch), including Alexa Fluor 405 donkey anti-rabbit (1:300), Alexa Fluor 488 donkey anti-rabbit (1:300), Alexa Fluor 488 donkey anti-guinea pig (1:300), Rhodamine Red-X donkey anti-rat (1:100), and Alexa Fluor 647 donkey anti-goat (1:300) were diluted in blocking buffer and applied at room temperature for 2 h. EdU labeling for newly generated cells was developed using the Click-iT EdU Alexa Fluor 647 Imaging Kit (catalog #C10340, Thermo Fisher Scientific). DAPI (1:1000) was added to secondary antibody solutions in some IHC panels.

### Immunohistochemistry analysis

Images for quantification were taken of the hippocampus at 10× magnification using the Axio Observer Fluorescent Microscope (Carl Zeiss Microscopy). All immunohistochemistry analyses were performed in coronal sections across the entire hippocampus, noting the distance from bregma for each slice. Measures were taken in the hippocampus of each hemisphere, and left and right values were averaged for each slice. Measures for each slice in a series were averaged to create a “whole” hippocampus average. Additionally, the hippocampus was divided along the dorsoventral axis into dorsal and ventral subregions, as in previous studies examining neurogenesis (Tanti et al., 2012; Yagi et al., 2019). Using coronal sections, the dorsal portion of the hippocampus generally included approximately four sections from approximately −1.06 to approximately −2.06 mm from bregma, and the ventral portion of the hippocampus included approximately three sections from approximately −2.92 to approximately −3.80 mm from bregma (Fig. 1*B*). All measurements were performed by an experimenter who was blinded to the identity of the treatment group from which sections came. Representative images are maximum intensity projections of *z*-stacks taken with a confocal microscope (model 880, Carl Zeiss Microscopy) using a 40× objective .

#### Quantification of neurogenesis-related measures

The number of Ki67^+^, DCX^+^, EdU^+^, EdU^+^/DCX^+^, and EdU^+^/NeuN^+^ cells in the entire dentate gyrus were manually counted on every sixth section throughout the entire hippocampus in ZEN software (blue edition, Carl Zeiss Microscopy).

#### Quantification of microglia-related measures

Iba1 and CD68 images were thresholded using ZEN (blue edition, Carl Zeiss Microscopy) and ImageJ (NIH) software. Regions of interest (ROIs) were drawn around the dentate gyrus and surrounding area of every sixth section using ImageJ (see Fig. 6) to quantify the average area covered by cells positive for each of these antibodies, as well as their colocalization. The “inner” (bordering the hilus) and “outer” ROIs of the dentate gyrus were created separately, as microglia activity in these subregions could be differentially regulating neurogenesis (Gemma and Bachstetter, 2013; Rodríguez-Iglesias et al., 2019).

### Statistical analysis

All data are expressed as the mean ± SEM. Data were analyzed using hypothesis testing by two-way ANOVA (factors: diet and sex). ANOVAs were followed by *post hoc* tests (Tukey’s method). Correlations were run separately for each sex to assess the relationships between metabolic outcomes and measures of neurogenesis and microglia in the subgranular zone of the dentate gyrus. Statistical significance was set at *p* < 0.05, and all statistical analyses were performed using GraphPad Prism version 8 software. Additionally, estimation statistics were performed using ESCI modules in Excel (https://thenewstatistics.com/itns/esci/) to report the mean percentage differences between groups, with 95% confidence intervals. Estimation statistics for significant group differences can be found in text, with all results reported in the Extended Data Fig. 2-1 and 6-1. section.

## Results

### High-fat diet results in metabolic impairments in male and female mice

Differences in metabolic responses to HF diet were assessed in males and females, and were previously reported for these mice (Salinero et al., 2018). All mice on an HF diet gained more weight compared with mice on a CON diet, and this was consistent across sexes. Of note, we found that males and females on an HF diet exhibited similar weight gain (mean ± SEM: males, 99.30 ± 5.36%; females, 87.90 ± 6.57%) and glucose intolerance [mean area under the curve (AUC) for GTT ± SEM: males, 57,496 ± 2436 mg/dl; females, 52,159 ± 1424 mg/dl] versus low fat-fed mice for weight gain (mean ± SEM: males, 26.40 ± 1.37%; females, 15.60 ± 2.28%) and glucose intolerance (mean AUC during GTT ± SEM: males, 39,535 ± 1360 mg/dl; females 29,527 ± 1340 mg/dl).

### High-fat diet decreases cell proliferation in the dorsal hippocampus in females only

**Figure 2.**
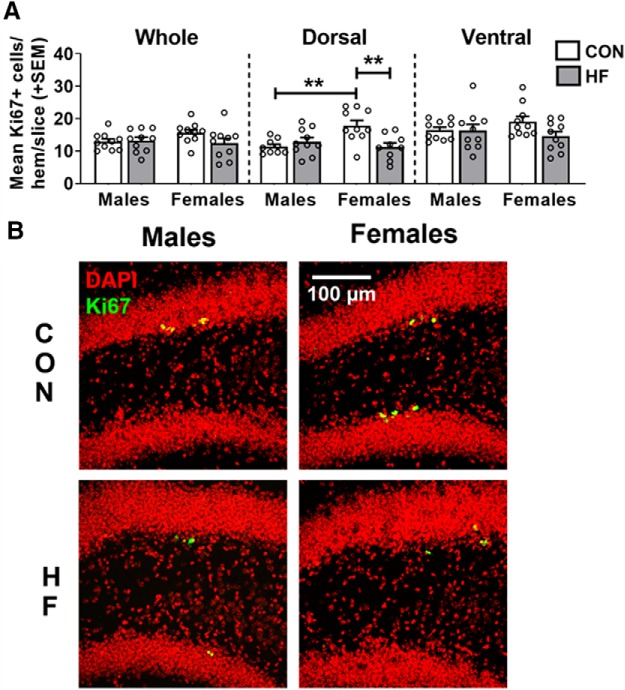
***A***, Cell proliferation in the dentate gyrus, as measured by the mean (±SEM) number of Ki67^+^ cells per hemisphere per 40 μm slice throughout the whole hippocampus, and in the dorsal and ventral subregions. On a control diet, females had a greater number of Ki67^+^ cells in the dorsal hippocampus compared with males, while HF diet reduced the number of Ki67^+^ cells in the dorsal hippocampus of females only. *N* = 10/group. **p < 0.01. ***B***, Representative images of Ki67 (green) and DAPI (red) immunostaining in the dorsal hippocampus. Estimation statistics for all neurogenesis-related measures can be seen in Extended Data Figure 2-1.

AHN occurs in a number of distinct and tightly regulated phases. In the current study, we assessed sex-dependent effects of HF diet on several stages of AHN in the SGZ of the dentate gyrus. AHN originates from quiescent neural progenitor cells, which give rise to transiently amplifying progenitor cells with high proliferative activity, expanding the number of cells capable of becoming newly born immature neurons (Kempermann et al., 2015). To assess these first stages of neurogenesis, the number of Ki67^+^ cells was counted in the dentate gyrus of the hippocampus (Fig. 2). Ki67 is an endogenous protein that is absent in quiescent cells but is expressed throughout all active phases of the cell cycle. Expression of this protein is not limited to cells destined to become neurons, therefore serving as a marker of cell proliferation (Kee et al., 2002). We counted the number of positive cells in sections throughout the entirety of the hippocampus and also quantified the number of cells in the dorsal and ventral subregions, which play a role in cognitive and affective processes, respectively (Fanselow and Dong, 2010; Kheirbek and Hen, 2011). While there were no significant effects of diet or sex in the whole hippocampus or the ventral subregion, group differences were apparent in the number of Ki67^+^ cells in the dorsal subregion. Within control mice, females had a greater number of Ki67^+^ cells compared with males in the dorsal hippocampus [*p* = 0.0060; mean difference, 57.01%; 95% CI (24.42%, 89.59%)]. While diet had no significant effect in males, HF females had fewer Ki67^+^ cells in the dorsal hippocampus compared with CON females [*p* = 0.0047; mean difference, −37.21%; 95% CI (−16.46%, −57.95%)]. The number of Ki67^+^ cells in HF females was similar to the number of Ki67^+^ cells in both male groups.

10.1523/ENEURO.0391-19.2019.f2-1 Figure 2-1 Estimation statistics were performed for neurogenesis-related measures, with data in the table representing the percentage differences between groups and 95% CI values. Values that are bolded/italicized/underlined and highlighted in gray were also found to be statistically significant via hypothesis testing (two-way ANOVA with Tukey’s *post hoc* test; α = 0.05). M CON, Male CON diet; M HF, male HF diet; F CON, females CON diet; F HF, female HF diet. Download Figure 2-1, XLSX file.

### High-fat diet decreases young/immature neurons in the dorsal hippocampus in females only

**Figure 3.**
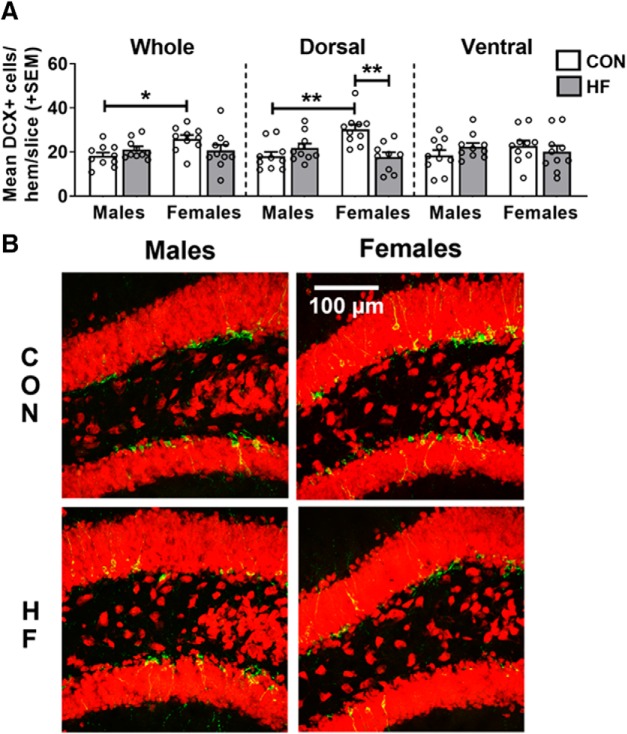
***A***, Number of neuroblasts/immature neurons in the dentate gyrus, as measured by the mean (±SEM) number of DCX^+^ cells per hemisphere per 40 μm slice throughout the whole hippocampus, and in the dorsal and ventral subregions. ***B***, On a CON diet, females had a greater number of DCX^+^ cells in the dorsal hippocampus compared with males, while an HF diet reduced the number of DCX^+^ cells in the dorsal hippocampus of females only. *N* = 10/group. *p < 0.05, **p < 0.01. ***B***, Representative images of DCX (green) and NeuN (red) immunostaining in the dorsal hippocampus.

Following the proliferative stages, newborn cells enter a postmitotic maturation phase. During this stage, some cells will have committed to a neuronal fate and begin establishing functional connections (Kempermann et al., 2015). To assess this differentiation/maturation phase, the number of DCX^+^ cells was counted in the dentate gyrus of the hippocampus (Fig. 3). Doublecortin (DCX) is an endogenous protein specific to cells committed to a neuronal fate that aids in the stabilization of microtubules during mitosis. DCX is expressed in new neurons starting at ∼4 d after birth until approximately ≥4 weeks in mice (Snyder et al., 2009), therefore serving as a marker of neuroblasts/immature neurons (von Bohlen und Halbach, 2011). Within CON mice, females had a greater number of DCX^+^ cells compared with males in the whole hippocampus [*p* = 0.0289; mean difference, 42.99%; 95% CI (13.18%, 72.80%)] and dorsal subregion (*p* = 0.0012; mean difference, 67.15%; 95% CI (34.05%, 100.26%)]. While diet had no significant effect in males, HF females had fewer DCX^+^ cells in the dorsal subregion compared with CON females (*p* = 0.0011; mean difference, −41.55%; 95% CI (−21.21%, −61.90%)]. The number of DCX^+^ cells in HF females was therefore similar to the number of DCX^+^ cells in both male groups. There were no significant effects of diet or sex in the ventral subregion of the hippocampus.

### Diet has no effect on maturation and survival of newly born neurons in the hippocampus

**Figure 4.**
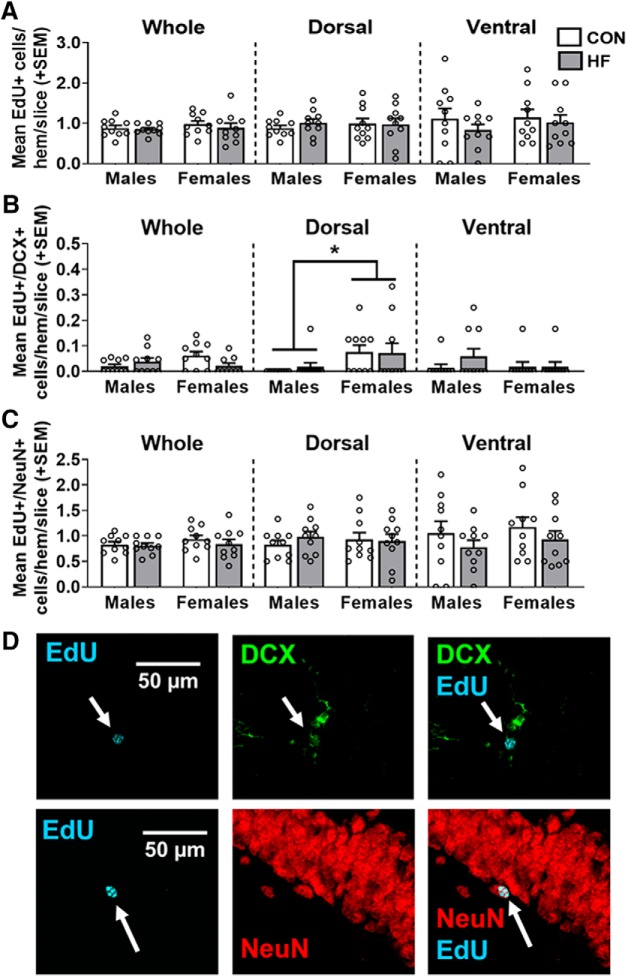
***A***, Survival of newly born cells in the dentate gyrus, as measured by the mean (±SEM) number of EdU^+^ cells 28 d post-EdU injection throughout the whole hippocampus and in the dorsal and ventral subregions. There were no group differences in any area measured. ***B***, Newly born cells in the dentate gyrus that differentiated into neuroblasts/immature neurons and survived 28 d post-EdU injection, were measured by the mean (±SEM) number of EdU^+^/DCX^+^ cells throughout the whole hippocampus and in the dorsal and ventral subregions. In the dorsal hippocampus, females had a greater number of EdU^+^/DCX^+^ cells compared with males, regardless of diet. ***C***, Newly born cells in the dentate gyrus that differentiated into mature neurons and survived 28 d post-EdU injection, were measured by the mean (±SEM) number of EdU^+^/NeuN^+^ cells throughout the whole hippocampus and in dorsal and ventral subregions. There were no group differences in any area measured. *N* = 10/group. *p < 0.05 main effect of sex. ***D***, Representative images of EdU (cyan) alone and colocalized with DCX (green) and NeuN (red).

Most newborn cells undergo apoptosis before reaching maturity (Kempermann et al., 2015); therefore, it is important to quantify not only how many new cells are produced, but also how many survive and become mature neurons. EdU is a thymidine analog, incorporated during DNA synthesis (S phase of mitosis), serving as an exogenous marker of cell proliferation (Buck et al., 2008). Mice were injected with EdU 4 weeks before euthanasia to determine the effects of sex and diet on newborn cell survival. EdU is not cell type-specific; therefore, we attempted to determine cell type by co-labeling with antibodies against DCX and NeuN. NeuN is an endogenous nuclear protein expressed by mature neurons (von Bohlen und Halbach, 2011). Maturation and survival of newly born neurons in the dentate gyrus was assessed by counting the number of cells that were EdU^+^ (newly born cells of any type), EdU^+^/DCX^+^ (newly born cells that began differentiating into neurons but remain in an immature phase), and EdU^+^/NeuN^+^ (newly born cells that differentiated into mature neurons; Fig. 4).

There was no significant effect of diet or sex on the number of EdU^+^ cells or EdU^+^/NeuN^+^ cells in the whole hippocampus, nor in the dorsal or ventral subregions. There were, however, significant group differences in the number of EdU^+^/DCX^+^ cells. Although the diet × sex interaction was significant in the whole hippocampus (*p* = 0.0325), no pairwise comparisons reached statistical significance. In the dorsal hippocampus, females had a greater number of EdU^+^/DCX^+^ cells compared with males overall (*p* = 0.016; mean difference, 778.31%; 95% CI (156.63%, 1400.00%)], while no effect of diet was seen in this subregion. There was no significant effect of diet or sex on the number of EdU^+^/DCX^+^ cells in the ventral hippocampus.

### Metabolic outcomes and neurogenesis are correlated in the dorsal hippocampus of females only

**Figure 5.**
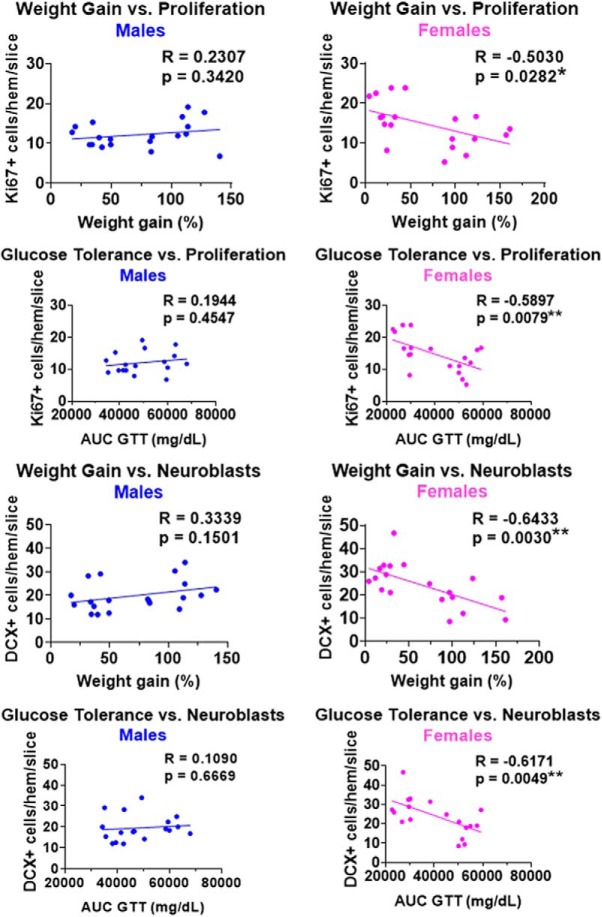
Correlations between metabolic outcomes [weight gain and glucose tolerance (AUC during GTT)] and neurogenesis measures [Ki67^+^ cells (proliferating cells) and DCX^+^ cells (neuroblasts/immature neurons)] in the dorsal hippocampus of males (left) and females (right). Mice on both control and high-fat diet are included in these plots. Note the significant inverse associations between metabolic outcomes and neurogenesis in females, but not males. *N* = 20/sex. *p < 0.05, **p < 0.01.

Pearson correlations were run within each sex to assess the relationships between metabolic outcomes and neurogenesis measures in the whole hippocampus, and dorsal and ventral subregions. Correlations between metabolic outcomes [body weight gain and glucose intolerance (AUC during GTT test)] and the number of Ki67^+^ cells (proliferating cells) and DCX^+^ cells (neuroblasts/immature neurons) in the dorsal subregion of the hippocampus are shown in Figure 5.

In males, there were no significant associations between metabolic outcomes and neurogenesis measures in the whole hippocampus or in either hippocampal subregion. However, significant relationships between these measures were observed in females, indicating that worse metabolic outcomes were predictive of more severe impairments in hippocampal neurogenesis. In the whole hippocampus, only one significant negative correlation was found in females. Greater glucose intolerance (higher AUC during GTT) was associated with fewer Ki67^+^ cells (*p* = 0.037); this relationship was similarly seen in the ventral subregion (*p* = 0.016) in females. Metabolic outcomes appeared to be most strongly associated with neurogenesis in the dorsal subregion of females, with both weight gain and AUC during GTT inversely correlated with the number of Ki67^+^ cells (weight gain, *p* = 0.028; AUC during GTT, *p* = 0.008) and DCX^+^ cells (weight gain, *p* = 0.003; AUC during GTT, *p* = 0.005). Metabolic outcomes were not significantly associated with the number of EdU^+^, EdU^+^/DCX^+^, or EdU^+^/NeuN^+^ cells in the whole hippocampus, or either hippocampal subregion, in males or females (data not shown).

### High-fat diet has sex-specific effects on microglia in the subgranular zone

**Figure 6.**
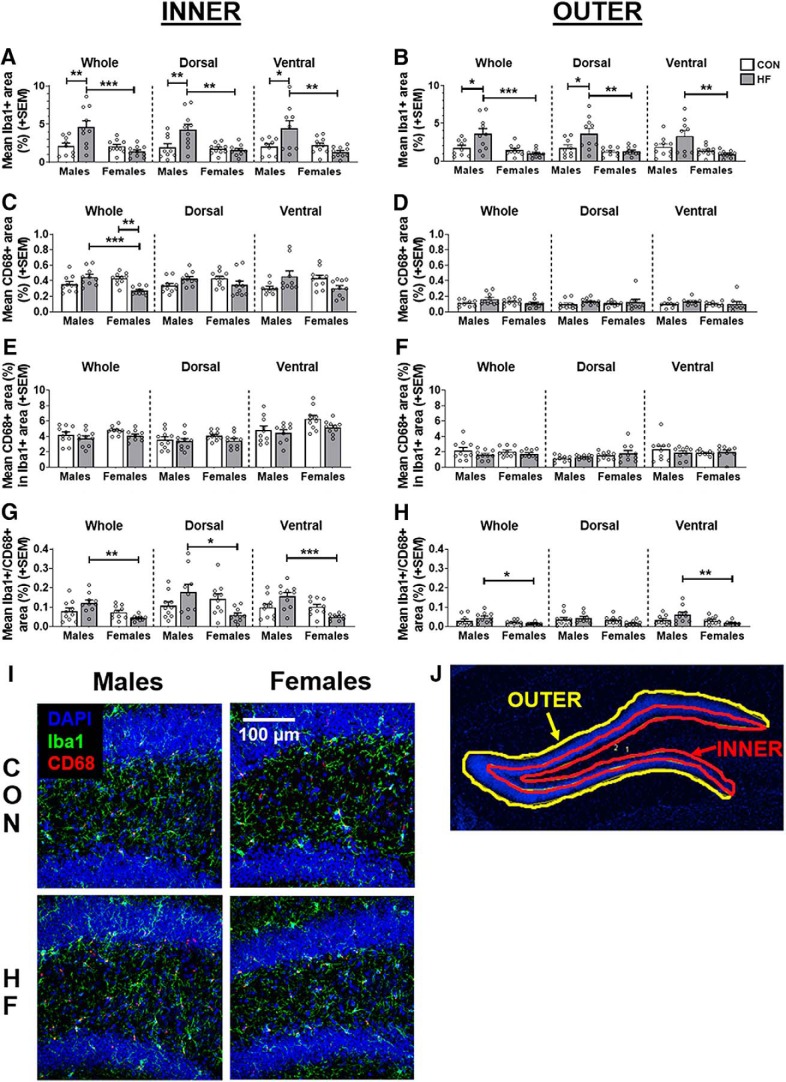
***A***, ***B***, Microglia density in the inner (***A***) and outer (***B***) dentate, as measured by the mean (±SEM) percentage area covered by Iba1^+^ stain throughout the whole hippocampus and in the dorsal and ventral subregions. ***C***, ***D***, Phagocytic activity in the inner (***C***) and outer (***D***) dentate, as measured by the mean (±SEM) percentage area covered by CD68^+^ stain throughout the whole hippocampus and in the dorsal and ventral subregions. ***E***, ***F***, Relative degree of microglial phagocytic activity in the inner (***E***) and outer (***F***) dentate, as measured by the mean (±SEM) percentage of Iba1^+^ area also positive for CD68 throughout the whole hippocampus and in the dorsal and ventral subregions. ***G***, ***H***, Absolute microglial phagocytic activity in the inner (***G***) and outer (***H***) dentate, as measured by the mean (±SEM) percentage area positive for colocalized Iba1 and CD68 throughout the whole hippocampus, and in the dorsal and ventral subregions. *N* = 10/group. *p < 0.05, **p < 0.01, ***p < 0.001. ***I***, Representative images of Iba1^+^ (green) and CD68^+^ (red) cells in the dorsal dentate gyrus. DAPI (blue) counterstaining was used to visualize nuclei, determine position relative to bregma, and create ROIs around the dentate gyrus. ***J***, Representative image of inner and outer ROIs of the dentate gyrus. See Figure 6-1 for estimation statistics performed for microglia-related measures.

Microglia play a multifaceted role in the neurogenic niche, regulating AHN by releasing growth factors and phagocytosing apoptotic newborn cells; additionally, microglia can adversely affect AHN under conditions that promote neuroinflammation (Aarum et al., 2003; Ekdahl et al., 2003; Morgan et al., 2004; Ziv and Schwartz, 2008; De Lucia et al., 2016; Kreisel et al., 2019). Therefore, microglia activity was assessed in the inner and outer portions of the dentate gyrus of the hippocampus (Fig. 6), as microglia activity in these subregions could be differentially regulating neurogenesis (Gemma and Bachstetter, 2013; Rodríguez-Iglesias et al., 2019). Neurogenesis occurs in the subgranular zone, which was included in the inner region of the dentate. We assessed the percentage positive area for Iba1 (microglia; Fig. 6*A*,*B*), CD68 (phagocytic marker; Fig. 6*C*,*D*), the percentage area of Iba1 also positive for CD68 (relative degree of microglial phagocytic activity; Fig. 6*E*,*F*), and the percentage area positive for colocalized Iba1 and CD68 (absolute microglial phagocytic activity; Fig. 6*G*,*H*).

10.1523/ENEURO.0391-19.2019.f6-1 Figure 6-1 Estimation statistics were performed for microglia-related measures, with data in the table representing the percentage differences between groups and 95% CI values. Values that are bolded/italicized/underlined and highlighted in gray were also found to be statistically significant via hypothesis testing (two-way ANOVA with Tukey’s *post hoc* test; α = 0.05). M CON, Male CON diet; M HF, male HF diet; F CON, females CON diet; F HF, female HF diet. Download Figure 6-1, XLSX file.

In both the inner and outer areas of the dentate gyrus, and across the whole hippocampus and within dorsal and ventral subregions, HF diet increased Iba1^+^ area in males only [*p* < 0.05 for all except outer ventral CON male vs HF male (*p* = 0.1137); mean differences ranging from 76.09% to 120.00%; Fig. 6*A*,*B*). This also resulted in a significant sex difference within HF diet-fed mice (males > females) in both the inner and outer areas of the dentate gyrus, across the whole hippocampus, and within the dorsal and ventral subregions (*p* < 0.0.05 for all; mean differences ranging from −63.14% to −73.12%; Fig. 6*A*,*B*).

Less pronounced trends, similar to Iba1^+^ area, were seen for absolute microglial phagocytic activity (percentage area positive for colocalized Iba1 and CD68; Fig. 6*G*,*H*). The area of positive staining was greater in HF males compared with HF females in the whole, dorsal, and ventral hippocampus for the inner dentate, and in the whole hippocampus and ventral subregion for the outer dentate (*p* < 0.05 for all; mean differences ranging from −63.43% to −70.61%).

In the inner portion of the dentate for the whole hippocampus, HF diet decreased the CD68^+^ area in females only [*p* = 0.0033; mean difference, −36.36%; 95% CI (−16.78%, −55.93%)]. Additionally, HF females had reduced CD68^+^ area compared with HF males [*p* = 0.0009; mean difference, −39.03%; 95% CI (−20.29%, −57.76%); Fig. 6*B*]. These trends were apparent but not significant in the dorsal and ventral subregions (*p* > 0.05 for all).

No differences between groups were observed for the percentage area of Iba1 also positive for CD68 (relative degree of microglial phagocytic activity; Fig. 6*C*).

### Microglia in the subgranular zone correlate with metabolic outcomes and neurogenesis in a sex- and region-specific manner

**Figure 7.**
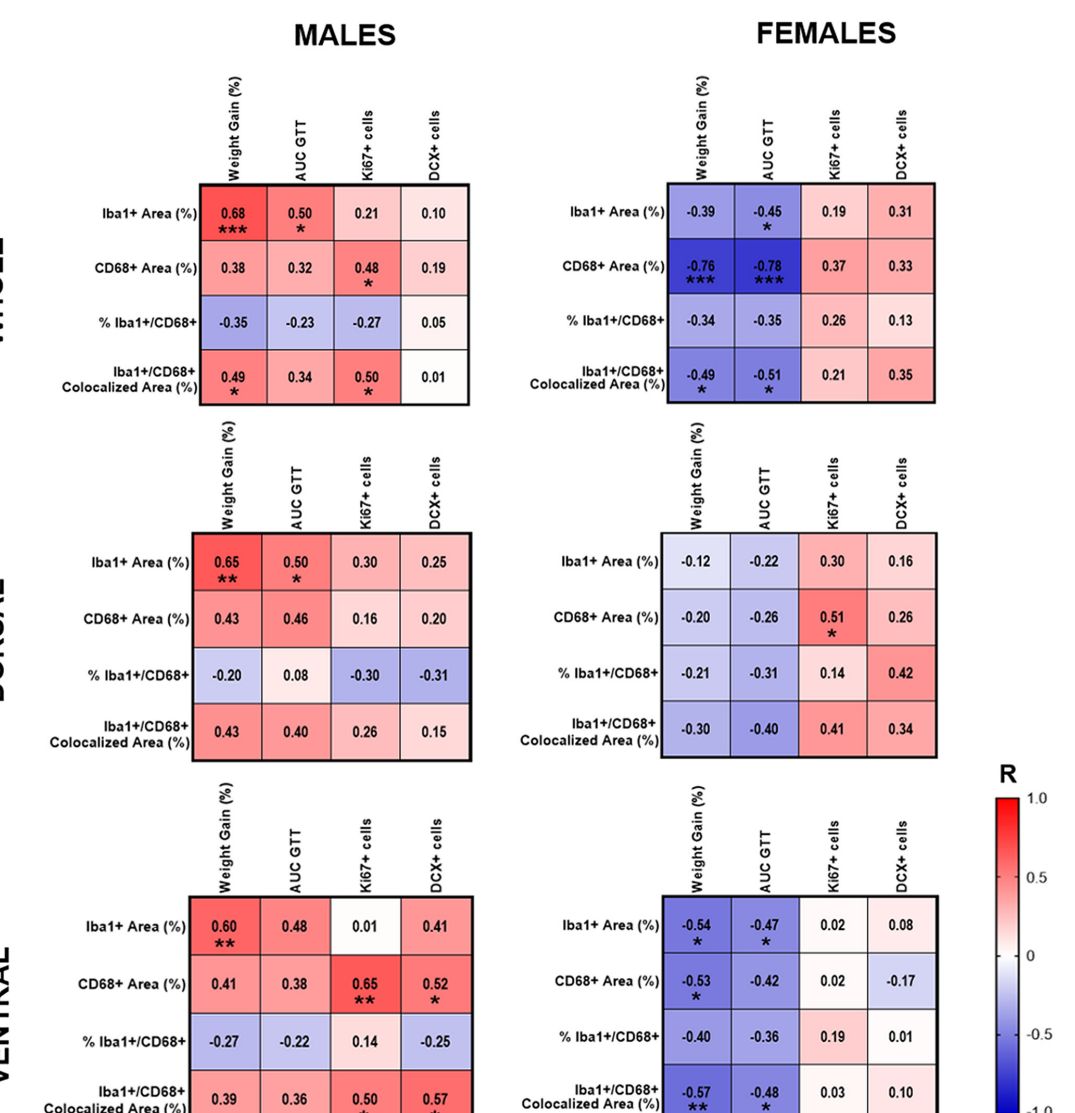
Correlations of microglia measures in the inner dentate with metabolic outcomes and neurogenesis in the whole hippocampus, and in dorsal and ventral subregions of male (left) and female (right) mice. Red indicates positive correlations, and blue indicates negative correlations, with intensity of the color indicative of the strength of the association. *p < 0.05, **p < 0.01.

Correlations between metabolic outcomes and microglia in the inner dentate gyrus were run separately by sex for the whole hippocampus and for dorsal and ventral subregions (Fig. 7). In males, metabolic outcomes (weight gain and AUC during GTT) were positively correlated with Iba1^+^ area in the whole, dorsal, and ventral subregions [p < 0.05 for all except vs AUC during GTT in ventral subregion (*p* = 0.062)]. There were also positive correlations in males between metabolic outcomes and CD68^+^ area, and metabolic outcomes with percentage area positive for colocalized Iba1 and CD68 (absolute microglial phagocytic activity) in the whole hippocampus and dorsal and ventral subregions (*r* = 0.32-0.49), though most of these relationships failed to reach statistical significance. In females, however, the opposite was seen, with metabolic outcomes negatively correlated with Iba1^+^ area, CD68^+^ area, and the percentage area positive for colocalized Iba1 and CD68 (absolute microglial phagocytic activity), particularly in the ventral subregion. There tended to be a negative correlation between metabolic outcomes and the percentage area of Iba1 also positive for CD68 (relative degree of microglial phagocytic activity) in both males and females, though none of these associations reached statistical significance.

Correlations were also run between microglia and neurogenesis measures in the dentate gyrus, again performed separately by sex for the whole hippocampus and for dorsal and ventral subregions (Fig. 7). In males, both CD68^+^ area and the percentage area positive for colocalized Iba1 and CD68 (absolute microglial phagocytic activity) were positively correlated with the number of Ki67^+^ cells in the whole hippocampus, and with both Ki67^+^ and DCX^+^ cells in the ventral hippocampus (*p* < 0.05 for all); weaker, nonsignificant trends of the same direction were seen in the dorsal hippocampus. In females, both CD68^+^ area and percentage area positive for colocalized Iba1 and CD68 (absolute microglial phagocytic activity) were positively correlated with the number of Ki67^+^ and DCX^+^ cells in the whole hippocampus and dorsal subregion, though most of these associations did not reach statistical significance; such correlations were not apparent in the ventral subregion in females (*p* > 0.05 for all). Microglia measures were not significantly associated with the number of EdU^+^, EdU^+^/DCX^+^, or EdU^+^/NeuN^+^ cells in the whole hippocampus or in either hippocampal subregion in males or females (data not shown).

## Discussion

The primary goal of this study was to determine whether there are sex differences in the effects of HF diet on AHN in mice. As previously reported, HF diet treatment from ∼2 to 6 months of age results in males and females developing a prediabetic phenotype (weight gain and glucose intolerance) to a similar degree of severity in both sexes (Salinero et al., 2018). The major finding of this study is that HF diet adversely affects AHN in a sex-, stage-, and subregion- specific manner. Specifically, we found that HF diet decreases cell proliferation and the number of young/immature neurons in the dorsal subregion of the hippocampus of females only. Moreover, within females, the degree of metabolic impairment was associated with greater neurogenic deficits. We also assessed the effects of HF diet on microglia activity in the dentate gyrus as a possible mechanism of sex-specific changes in neurogenesis. We uncovered sex-specific effects of HF diet on microglia, as well as relationships between microglia activity and neurogenesis, which suggest that an increased presence of microglia may be protective against decreases in neurogenesis in the presence of metabolic disease.

### Sex differences in neurogenesis in metabolically healthy mice

We found that in metabolically healthy animals fed a control diet, females exhibit greater levels of cell proliferation (Ki67^+^ cells) and more neuroblasts/immature neurons (DCX^+^ cells). These findings are in agreement with previous studies reporting that female rodents have greater proliferation, which may depend on season or cycle of the estrus phase, compared with males (Galea and McEwen, 1999; Tanapat et al., 1999; Spritzer et al., 2017), though a previous study found that male rats had a greater number of immature neurons compared with females (Hillerer et al., 2013). Observed sex differences may be mediated at least in part by sex hormones, as estradiol treatment has been shown to increase proliferation in both intact and ovariectomized female rats (Tanapat et al., 1999; Duarte-Guterman et al., 2015). Additionally, proliferation varies throughout the estrus cycle, with levels of proliferation greatest during the proestrus phase when estradiol levels are highest (Tanapat et al., 1999), though some have failed to show sex differences or estrus cycle effects on proliferation in mice (Amrein et al., 2004; Lagace et al., 2007). As we did not monitor the estrus cycle, we cannot speculate whether this played a role in the observed increase in cell proliferation and the number of neuroblasts/immature neurons in control diet-fed females compared with males.

While the number of young/immature neurons was greater in females compared with males, there was no difference in the number of surviving mature neurons (EdU^+^/NeuN^+^ cells) in the dentate gyrus. Lack of sex differences in new neuron survival has been reported in previous work in rodents (Tanapat et al., 1999; Barker and Galea, 2008; Spritzer et al., 2017). These findings could be explained either by a higher rate of death of newly born cells or by neurons remaining in an immature state in females. The former possibility is supported by previous findings that female rats exhibit greater degeneration (pyknotic cells) in the SGZ and granule cell layer compared with males throughout most of the estrus cycle, and no sex difference in BrdU-labeled cell survival or total number of granule neurons in the dentate gyrus (Tanapat et al., 1999). The latter possibility is supported by our findings that females (regardless of diet) exhibited a greater number of EdU^+^/DCX^+^ cells in the dentate gyrus compared with males, representative of 4-week-old cells that still remained in a neuroblast/immature neuron state. As neurons reach maturation, they trade expression of DCX for mature neuron markers such as NeuN. It was previously shown in mice that at 4 weeks post-BrdU injection (time equivalent to our observed time point), ∼50% of the cells are NeuN^+^ and ∼ 30% are DCX^+^; however, this study was performed in male mice only (Snyder et al., 2009). It is possible that maturation of neurons may occur more slowly in females compared with males, as suggested by a recent study in rats (Yagi et al., 2019). There is evidence to suggest that immature neurons may be functionally relevant (see discussion below), and their contribution to observed sex differences in hippocampal-dependent cognitive strategies and performance (Yagi and Galea, 2019) warrant future study.

### Sex differences in the effects of high-fat diet/prediabetes on adult hippocampal neurogenesis

The current study found that an HF diet decreased cell proliferation and the number of neuroblasts/immature neurons in the dentate gyrus of females, while males were unaffected by diet. Additionally, there were strong negative associations between metabolic dysfunction (weight gain and glucose intolerance) and these measures of neurogenesis in females. These correlations were not seen in males, as expected, due to lack of diet effect. Previous studies have found that high-fat diet and metabolic disease such as obesity and diabetes disturb several aspects of neurogenesis. For example, mice administered a high-fat diet show impaired neurogenesis and decreased BDNF, as well as increased lipid peroxidation (Park et al., 2010). Similarly, administration of a high-fat diet for 8 weeks beginning at 6 weeks of age to male C57BL6/J mice resulted in increased body mass and fasting blood glucose levels, in addition to reduced BDNF and number of Ki67^+^, DCX^+^, and BrdU^+^ cells in the dentate gyrus of the hippocampus (Nam et al., 2017). It is important to note that initiating the diet early (6 weeks of age) in males elicits a type 2 diabetic phenotype, in contrast to our current study in which diet was initiated at 8 weeks of age and resulted in a prediabetic phenotype. Genetic and pharmacological rodent models of diabetes also demonstrate impaired neurogenesis, including reductions in Ki67 and DCX in the dentate (Jackson-Guilford et al., 2000; Yi et al., 2009; Chung et al., 2015; Yau et al., 2018). Our findings contrast with those of one of the first studies on HF diet in juvenile Sprague Dawley rats, which found that 4 weeks of eating a high-fat diet reduced hippocampal neurogenesis (number of BrdU^+^ cells 2 weeks postinjection) by ∼40% in males in the absence of obesity; however, while an HF diet increased weight gain and adiposity in females, no effect on neurogenesis was apparent (Lindqvist et al., 2006). Only weight gain and fat mass were assessed in these rats, while their glucose tolerance/diabetic status is unknown. The negative influences of an HF diet on Ki67 and DCX have been demonstrated in male mice of other strains (Hwang et al., 2008). Other studies using male C57BL/6J mice failed to show any differences in DCX when HF diet was started in adulthood (12 weeks of age; Boitard et al., 2012), similar to our own findings. Sex differences in the susceptibility of adult hippocampal neurogenesis with other exogenous factors, such as stress, learning, and exercise, have been noted (Falconer and Galea, 2003; Dalla et al., 2009; Chow et al., 2013; Hillerer et al., 2013; Yagi et al., 2016; Cahill et al., 2018). For example, one study found that chronic restraint stress during adolescence was found to attenuate cell proliferation and survival in adult female but not male rats (Barha et al., 2011). The extent to which these sex differences are mediated by sex hormones remains to be determined. As discussed, estradiol and estrus cycle phase affect neurogenesis (Tanapat et al., 1999; Duarte-Guterman et al., 2015), and diet induced-obese female mice have been shown to exhibit estrus cycle abnormalities and altered levels of sex hormones and enzymes involved in steroidogenesis (Lai et al., 2014; Wu et al., 2014; Hohos et al., 2018). Recent evidence also suggests that estrogen receptor (ER)-α and/or ER-β play a role in type 2 diabetes-induced impairments in neurogenesis and cognitive function in mice (Tang et al., 2019).

The hippocampus is a heterogeneous structure, with the dorsal subregion (septal pole) functionally distinct from the ventral subregion (temporal pole), corresponding to the human posterior and anterior portions of the hippocampus, respectively. The dorsal hippocampus is implicated in cognitive processes, including spatial learning and memory, while the ventral hippocampus is involved in affective processes, such as emotion regulation and stress reactivity (O'Leary and Cryan, 2014). In addition to behavior studies, there is evidence for this heterogeneity based on gene expression and anatomic connectivity (O'Leary and Cryan, 2014). While the dorsal hippocampus is connected to brain regions involved in visuospatial processing, spatial navigation/exploration, and spatial memory (e.g., anterior cingulate cortex, retrosplenial cortex, mammillary nuclei), the ventral hippocampus appears to communicate with regions involved in neuroendocrine function, stress, anxiety, reward, motivation, and executive function (e.g., hypothalamus, amygdala, nucleus accumbens, and medial prefrontal cortex; O'Leary and Cryan, 2014). Despite the fact that the segregation of the dorsal and ventral subregions has been generally accepted, few have assessed neurogenesis in these two subregions separately. However, previous studies that have examined the effects of environmental factors on neurogenesis in the dorsal and ventral subregions separately have often found that manipulations preferentially affect facets of neurogenesis in one subregion versus the other (Tanti et al., 2012, 2013; O'Leary and Cryan, 2014). For example, unpredictable chronic mild stress decreased proliferation and neurogenesis preferentially in the ventral hippocampus, while environmental enrichment increased proliferation in both subdivisions but increased neurogenesis in the dorsal hippocampus only (Tanti et al., 2012). Interestingly, the current study suggests that a high-fat diet and the resulting metabolic dysfunction preferentially disturb neurogenic processes in the dorsal subregion of the hippocampus, and that this occurs in females only. Our findings of HF diet-induced disruption of neurogenic processes in the dorsal hippocampus are in agreement with studies that have shown that HF diet impairs cognitive function that is dependent on this subregion of the hippocampus (Cordner and Tamashiro, 2015), such as performance on the latent cue preference task or the conditioned cue preference task in a multiple-cue environment, while other forms of learning are spared (Stouffer et al., 2015).

While we did not observe an effect of diet on the long-term (4 week) survival of newly born neurons in either sex, high-fat diet/metabolic dysfunction-induced decreases in cell proliferation and, particularly, the number of young/immature neurons may be functionally significant. It has been shown that new cells in the dentate gyrus can extend their axons into the CA3 region as early as 4–10 days post-BrdU incorporation (Hastings and Gould, 1999). This suggests that, even at an immature stage, these cells may have a functional impact. This is further supported by evidence that young/immature neurons contribute a form of LTP in the hippocampus that is distinct (lower threshold, longer-lasting, weaker GABAergic inhibition) from that supported by more mature neurons (Wang et al., 2000; Snyder et al., 2001). These neuroblasts/immature neurons are implicated in long-term spatial memory, while more mature neurons are hypothesized to support acquisition and short-term retention (Snyder et al., 2005). Therefore, the effects of diet and metabolic dysfunction on the production and/or maintenance of immature neurons may have significant implications for hippocampal structure and function in females. We are currently investigating whether HF diet/prediabetes causes greater impairments in dorsal hippocampus-dependent spatial memory tasks (e.g., Morris water maze) in females compared with males in mouse models of healthy aging and dementia.

### Sex differences in the effects of high-fat diet/prediabetes on microglia activity and relation to neurogenesis

We sought to determine whether the adverse effects of HF diet on neurogenesis in females could be due to increased neuroinflammation mediated by microglia, the resident immune cell of the brain. We hypothesized that this could be the case based on previous findings that high-fat diet/metabolic disease are associated with increased inflammation, including microglia activity in the hippocampus (Kang et al., 2016; Ledreux et al., 2016; Yu et al., 2019), and that microglial-associated inflammation (i.e., via release of inflammatory cytokines such as IL-1β, TNF-α, and IL-6) impairs AHN in rodents (Ekdahl et al., 2003; Monje et al., 2003). Our results indicate that while there were no sex differences in microglia measures in mice on a control diet, we uncovered sex-specific effects of HF diet on microglia in the dentate gyrus. We focused on the inner dentate gyrus, which included the subgranular zone, as this is the region where proliferating cells (Ki67^+^) and young/immature neurons (DCX^+^) are found, and these were the cells affected by an HF diet. However, many findings were similar though less robust in the outer dentate. We found that HF diet increased the area of the dentate gyrus covered by microglia (Iba1^+^), an effect that was consistent across the entire region; however, this occurred in males only. There was no increase in microglial measures in females on an HF diet; in fact, trends fell in the opposite direction. Sex differences are also demonstrated by positive correlations between metabolic outcomes and microglia measures in males, and negative relationships between these measures in females.

In addition to microglia-mediated neuroinflammation having adverse effects on AHN, there are several mechanisms by which microglia support AHN (Ziv and Schwartz, 2008), enhancing proliferation, neuronal differentiation, migration, and survival (Aarum et al., 2003; Morgan et al., 2004; Walton et al., 2006). In fact, microglia appear to be essential for the survival of neuroblasts (Kreisel et al., 2019), while inhibiting microglia activity with minocycline treatment attenuates AHN (Ziv et al., 2006). Microglia produce neuroprotective factors such as FGF-2, IGF-1, and BDNF that likely contribute to their proneurogenic effects (Ziv and Schwartz, 2008). Additionally, interventions like environmental enrichment, which have been shown to enhance hippocampal BDNF, increase microglial activation and Iba1^+^ expression in the dentate (but not in CA1 or CA3), suggesting that microglia activity may play a positive role in supporting neurogenic processes (Williamson et al., 2012). Moreover, enhanced neurogenesis observed in some cases following brain insult or disease may be mediated by activated microglia (Liu et al., 1998; Ogita et al., 2012; De Lucia et al., 2016). It is possible that the enhanced microglia markers observed in HF-fed males is neuroprotective, as HF males do not experience detrimental effects of diet on neurogenesis. HF diet-induced deficits in cell proliferation and young/immature neurons in females are not accompanied by similarly increased measures of microglia markers; in fact, the opposite is observed.

Microglia also play a role in phagocytosing apoptotic cells in the dentate gyrus, as many proliferating and young neurons do not survive into maturity; this process protects against the spilling of toxic cellular contents from apoptotic cells (Gemma and Bachstetter, 2013; Sato, 2015). Negative correlations between metabolic outcomes with microglia and phagocytic markers in females, associated with dysregulated neurogenesis, may therefore represent sex-specific impairments in microglia function. Impairments of hippocampal microglia phagocytic capability have been demonstrated in response to other environmental influences (e.g., inescapable stress), which appear to be more severe in females compared with males (Fonken et al., 2018). There are also known sex differences across normal development and aging in microglia in several brain structures, as well as sex-mediated expression of cytokines and chemokines in regions such as the cortex and hippocampus (Schwarz and Bilbo, 2012; Schwarz et al., 2012; Kopec et al., 2018). However, further investigation is necessary to investigate sex differences in the role of microglia in AHN under pathophysiological conditions, including metabolic disease.

### Conclusions

In the current study, we found that chronic administration of a high-fat diet from ∼2 to 6 months of age produces striking sex differences in its consequences on adult hippocampal neurogenesis. Cell proliferation, neuroblasts, and new neuron maturation and survival were unaffected by diet in males. In females, however, high-fat diet significantly reduced cell proliferation and the number of young/immature neurons. Additionally, metabolic dysfunction was correlated with impaired neurogenesis in females only. These effects of diet and metabolic impairment on neurogenesis were robust in the dorsal hippocampus, which plays a role in cognitive functions like learning and memory (Fanselow and Dong, 2010; Kheirbek and Hen, 2011). It was previously reported that this diet regimen results in a similar prediabetic phenotype (increased weight gain and glucose intolerance) in male and female C57BL6/J mice (Salinero et al., 2018). Therefore, the observed sex-specific effects of HF diet on neurogenesis are not likely attributable to sex differences in metabolic susceptibility to the diet. It was also recently reported that the metabolic effects of HF diet vary by age and sex, such that males experience greater metabolic impairment compared with females when diet begins as juveniles (6 weeks of age), but females experience greater weight gain and glucose intolerance compared with males when diet begins at middle age (∼8.5 months; Salinero et al., 2018). It would be of interest to determine whether sex differences in AHN vary by age of diet onset as well, and how this relates to age- and sex-dependent effects of HF diet on metabolic outcomes.

Evidence suggests that detrimental diet effects may be reversible, as switching from the high-fat to the control diet is capable of restoring neurogenesis in rodents; however, this study was performed in male rats only (Boitard et al., 2016). Interventions that improve metabolic outcomes, such as physical exercise and caloric restriction, promote neurogenesis and gliogenesis, increase growth factors and synaptic plasticity in the hippocampus, and improve cognitive performance (Lee et al., 2002; Bondolfi et al., 2004; Park and Lee, 2011; Kaptan et al., 2015; Chatterjee et al., 2016; Klein et al., 2016). Moreover, treatment with medications traditionally used to treat type 2 diabetes increases adult hippocampal neurogenesis in HF diet-fed rodents (Hamilton et al., 2011; Porter et al., 2013; Pathak et al., 2015; Tanokashira et al., 2018). If adult hippocampal neurogenesis serves to preserve cognitive function during aging and neuropathology (e.g., AD pathology), but metabolic disease impairs neurogenesis, those with metabolic disease would be at increased risk for cognitive decline, particularly in women. This is of great clinical relevance, especially given the sex bias in dementia and AD particularly (Hebert et al., 2013; Beam et al., 2018; Buckley et al., 2019). AD medications promote neurogenesis (Jin et al., 2006), and enhancing neurogenesis with genetic (Richetin et al., 2015), pharmacological (Blanchard et al., 2010), and behavioral (Valero et al., 2011; Tapia-Rojas et al., 2016) interventions rescues cognitive performance in AD mouse models. Therefore, targeting metabolic disease or preventing/reversing its detrimental effects on hippocampal neurogenesis could reduce the burden of dementia in clinical populations, particularly women.

## Synthesis

Reviewing Editor: Margaret McCarthy, Univ of Maryland School of Medicine Decisions are customarily a result of the Reviewing Editor and the peer reviewers coming together and discussing their recommendations until a consensus is reached. When revisions are invited, a fact-based synthesis statement explaining their decision and outlining what is needed to prepare a revision will be listed below. The following reviewer(s) agreed to reveal their identity: Pelin Cengiz, Jaylyn Waddell.

This manuscript finds that a high fat diet reduces cell proliferation in the dorsal hippocampus of female mice but not male mice. There are conflicting reports that this paper references. Age of the mouse is cited as a potential contributor to this discrepancy. Neither survival of cells nor differentiation of the cells is impacted. Metabolic outcomes, i.e., glucose intolerance, was correlated with cell genesis in females only, but this would be the only possible group to exhibit such a correlation, since males are unchanged by diet. Microglia staining revealed higher numbers induced by high fat diet only in males.

These findings are novel, and are appropriately discussed in light of the existing literature.

The statistical approach you have used is deemed appropriate by both reviewers, however I encourage you to consider also reporting estimation statistics that more accurately reflect data distribution and effect sizes. You can find further information on how to do so here: https://doi.org/10.1523/ENEURO.0205-19.2019

The reviewers have the following comments:

1. Normal diet versus low fat diet: The reported and universally considered normal diet of adult mice consists of 10% fat and so the current study should not refer to it as a low fat diet but a normal diet for mice (see Winzell MS, et al. High-fat diet fed mouse. Neurosci Letter 2010 or Boitard C, et al. Hippocampus 2012).

2. How is the “metabolic outcome” or “metabolic syndrome” defined? Is it the percent weight gain and glucose intolerance? What percentage of weight gain or the AUC GTT defined as mice having metabolic syndrome? What is the percentage of the weight gain among treatment groups? In Figure 5, correlations between the percent weight gain/AUC GTT with Ki67+/DCX+ cells are shown. There is large variability in weight gain, anywhere between 0-150% weight gain is reported. Were all the mice, both normal and high fat diet, included in this graph? Please say so in the figure legend if true. More details in methodology are required in order to show that the mouse fed with high fat diet clearly show the phenotype of a metabolic syndrome.

3. The data presented is exclusively IHC and quantified using different neurogenesis markers, including Ki67 and DCX. The quantification of these Ki67+, DCX+ cells, iba1, etc...need more detail. Under IHC analysis section, authors say that they analyzed all of the slices obtained through hippocampus but then they say they analyzed every 6th section though hippocampus using image J. Adult mouse hippocampus when the brains are sectioned 40 microns should have on average 40-45 slices. Does that mean, authors analyzed 6-7 slices per mouse?

4. The authors find that females exhibit more Edu+ cells that co-express DCX 4 weeks after EDU injection. The state that this might reflect the fact that the cells get “stuck” in an immature phase. Though they go on to point out that it could be the immature state cells have functional effects, as they may extend neurites much earlier and have been implicated in spatial working memory, the term “stuck” in this context is used more than once. This reviewer thinks this should be removed. It is the case in both low fat and high fat diet female mice, suggesting it isn't pathological. “Stuck” implies otherwise.

5. The suggestion that microglia are protective in the male brain doesn't seem well supported by the correlation matrices presented in Figure 7. Using the dorsal hippocampus as an example, %Iba correlated similarly between males and females. CD68 positively correlates with Ki67 in females, and only females exhibit any change based on diet. The sex difference seems to be driven by the ventral hippocampus, but the effect of diet was found only in the female dorsal hippocampus. The authors might consider clarifying these results. To propose that microglia are protective in male brain would assume that if they were to lose cells due to diet, they would be lost in the ventral hippocampus. This seems to be a bit of a stretch. The fact that microglia numbers are different and correlate in a sexually divergent fashion is interesting, but the interpretation is confusing.

6. Metabolic outcomes, i.e., glucose intolerance, was correlated with cell genesis in females only, but this would be the only possible group to exhibit such a correlation, since males are unchanged by diet.

7. In figure 2, the Ki67 signal is very weak, it is hard to see the representative Ki67 staining. Using a higher magnification or an inset where it shows the Ki67 staining in the nucleus would be nice.

8. In figure 3, the DCX stained cells are hard to see. Staining suggests that DCX stained mostly the nucleus. However, since DCX stains the microtubular associated proteins in neuroblasts, it stains the dendritic processes. It is very hard to see whether DCX staining is present in microtubules or not in 10x magnification. Higher magnifications or better representative images are needed.

9. Authors should consider using the Stereo Investigator program to count co-localized cells for more powerful conclusions instead of semi-quantitative quantifications.

10. Figure legends need more detailed explanations of the figures.

10, The extended data present data that are used in correlations presented in the main document. Though not critical, readers may be interested to see the magnitude of the effects, as the diverge between males and females

## Author Response

We thank the reviewers for their thoughtful comments. Our responses are shown below in blue. Changes to the manuscript are shown in red.

Editor comments:

The statistical approach you have used is deemed appropriate by both reviewers, however I encourage you to consider also reporting estimation statistics that more accurately reflect data distribution and effect sizes.

As suggested, estimation statistics were performed using ESCI modules in excel (https://thenewstatistics.com/itns/esci/) to report mean percent differences between groups, with 95% confidence intervals. This has also been put into table format in extended data. Relevant information on these statistics have been added to the “Statistical analysis” portion of the Methods section.

The reviewers had the following comments:

1. Normal diet versus low fat diet: The reported and universally considered normal diet of adult mice consists of 10% fat and so the current study should not refer to it as a low fat diet but a normal diet for mice (see Winzell MS, et al. High-fat diet fed mouse. Neurosci Letter 2010 or Boitard C, et al. Hippocampus 2012).

The authors have changed “low fat diet” to “control diet” throughout the text and in the figures.

2. How is the “metabolic outcome” or “metabolic syndrome” defined? Is it the percent weight gain and glucose intolerance? What percentage of weight gain or the AUC GTT defined as mice having metabolic syndrome? What is the percentage of the weight gain among treatment groups? In Figure 5, correlations between the percent weight gain/AUC GTT with Ki67+/DCX+ cells are shown. There is large variability in weight gain, anywhere between 0-150% weight gain is reported. Were all the mice, both normal and high fat diet, included in this graph? Please say so in the figure legend if true. More details in methodology are required in order to show that the mouse fed with high fat diet clearly show the phenotype of a metabolic syndrome.

The authors agree it is imperative to report metabolic outcomes for each group to ensure that the HF diet actually results in a “metabolic syndrome” phenotype. We did not include direct references to the metabolic outcomes of these mice, as they were previously published, and we were being careful not to reveal our identities to maintain the double-blindness of the review process. In this round of revisions, we have made explicit references to our previous work in these mice in the “unblinded” version of the manuscript. We have also added a new section to the results referencing our prior paper, and also reporting means and SEMs needed to demonstrate that HF diet resulted in more weight gain and glucose intolerance compared to control diet mice, and to a similar degree in males and females. Please see new first paragraph of the results section. 2) We have added to the figure legend for Figure 5: “Mice on both control and high fat diet are included in these plots.”

3. The data presented is exclusively IHC and quantified using different neurogenesis markers, including Ki67 and DCX. The quantification of these Ki67+, DCX+ cells, iba1, etc...need more detail. Under IHC analysis section, authors say that they analyzed all of the slices obtained through hippocampus but then they say they analyzed every 6th section though hippocampus using image J. Adult mouse hippocampus when the brains are sectioned 40 microns should have on average 40-45 slices. Does that mean, authors analyzed 6-7 slices per mouse?

We cut brains into 6 series, with one series used for each panel of IHC. Hippocampal sections in the range of ~-1.06 to ~-3.80 were stained and analyzed. With a distance of about 2.74mm of hippocampus cut at 40um, this would theoretically create ~68.5 sections in total. Cutting into 6 series theoretically then creates ~11 sections per series (range usually 10-12). All sections in each set were analyzed for the “whole” hippocampus measure. The “dorsal” hippocampus ranging from ~-1.06 to ~-2.06 has ~4 sections per series, and the “ventral” hippocampus from ~-2.92 to ~-3.80 has ~3 sections per series.

To clarify in the text, the following has been added to the “Immunohistochemistry” portion of the Methods: “Hippocampal sections (ranging from ~-1.06 to ~-3.80 mm from Bregma) from one of these series were used for each panel of immunohistochemistry (IHC), resulting in ~10-12 sections analyzed throughout the entirety of the hippocampus.”

Additionally, we have revised in the “Immunohistochemistry analysis” portion of the Methods: “Measures for each slice in a series were averaged to create a ‘whole‘ hippocampus average” “Using coronal sections, the dorsal portion of the hippocampus generally included ~4 sections from ~-1.06 to ~-2.06 mm from Bregma, and the ventral portion of the hippocampus included ~3 sections from ~-2.92 to ~-3.80 mm from Bregma (Figure 1B).”

4. The authors find that females exhibit more Edu+ cells that co-express DCX 4 weeks after EDU injection. The state that this might reflect the fact that the cells get ‘stuck’ in an immature phase. Though they go on to point out that it could be the immature state cells have functional effects, as they may extend neurites much earlier and have been implicated in spatial working memory, the term “stuck” in this context is used more than once. This reviewer thinks this should be removed. It is the case in both low fat and high fat diet female mice, suggesting it isn't pathological. ‘Stuck’ implies otherwise.

We agree and thank the reviewer for this suggestion to avoid possibly mislabeling this as a pathological finding. We have edited the Discussion in two places to now read:

“Maturation and survival of newly born neurons in the dentate gyrus was assessed by counting the number of cells that were EdU+ (newly born cells of any type), EdU+/DCX+ (newly born cells that began differentiating into neurons but remain in an immature phase) and EdU+/NeuN+ (newly born cells that differentiated into mature neurons) (Figure 4).”

“These findings could be explained by either a higher rate of death of newly born cells, or neurons remaining in an immature state, in females.”

5. The suggestion that microglia are protective in the male brain doesn't seem well supported by the correlation matrices presented in Figure 7. Using the dorsal hippocampus as an example, %Iba correlated similarly between males and females. CD68 positively correlates with Ki67 in females, and only females exhibit any change based on diet. The sex difference seems to be driven by the ventral hippocampus, but the effect of diet was found only in the female dorsal hippocampus. The authors might consider clarifying these results. To propose that microglia are protective in male brain would assume that if they were to lose cells due to diet, they would be lost in the ventral hippocampus. This seems to be a bit of a stretch. The fact that microglia numbers are different and correlate in a sexually divergent fashion is interesting, but the interpretation is confusing.

The authors agree that the data and interpretation of microglia findings were a bit unclear in the original version. We have attempted to remove interpretations that are not clearly supported, and additionally discussed possible impairment of microglia activity in females resulting from HF diet, as has been seen in response to other stressors, as follows:

“Microglia also play a role in phagocytosing apoptotic cells in the dentate gyrus, as many proliferating and young neurons do not survive into maturity; this process protects against the spilling of toxic cellular contents from apoptotic cells (Gemma and Bachstetter, 2013; Sato, 2015). Negative correlations between metabolic outcomes with microglia and phagocytic markers in females, associated with dysregulated neurogenesis, may therefore represent sex-specific impairments in microglia function. Impairments of hippocampal microglia phagocytic capability has been demonstrated in response to other environmental influences (e.g. inescapable stress), which appear to be more severe in females compared to males (Fonken et al., 2018). There are also known sex differences across normal development and aging in microglia in several brain structures, as well as sex-mediated expression of cytokines and chemokines in regions such as the cortex and hippocampus (Schwarz and Bilbo, 2012; Schwarz et al., 2012; Kopec et al., 2018). However, further investigation is necessary to investigate sex differences in the role of microglia in AHN under pathophysiological conditions, including metabolic disease.”

6. Metabolic outcomes, i.e., glucose intolerance, was correlated with cell genesis in females only, but this would be the only possible group to exhibit such a correlation, since males are unchanged by diet.

The authors acknowledge the validity of this comment, and have modified the text accordingly in the discussion as follows: “The current study found that HF diet decreased cell proliferation and the number of neuroblasts/immature neurons in the dentate gyrus of females, while males were unaffected by diet. Additionally, there were strong negative associations between metabolic dysfunction (weight gain and glucose intolerance) and these measures of neurogenesis in females. These correlations were not seen in males, as expected, due to lack of diet effect.”

7. In figure 2, the Ki67 signal is very weak, it is hard to see the representative Ki67 staining. Using a higher magnification or an inset where it shows the Ki67 staining in the nucleus would be nice.

Our original representative images taken at 10x have been replaced by higher magnification (40x) maximum intensity projection images generated from z-stack images using a confocal microscope. This has also been updated in the Methods section.

8. In figure 3, the DCX stained cells are hard to see. Staining suggests that DCX stained mostly the nucleus. However, since DCX stains the microtubular associated proteins in neuroblasts, it stains the dendritic processes. It is very hard to see whether DCX staining is present in microtubules or not in 10x magnification. Higher magnifications or better representative images are needed.

Our original representative images taken at 10x have been replaced by higher magnification (40x) maximum intensity projection images generated from z-stack images using a confocal microscope. This has also been updated in the Methods section.

9. Authors should consider using the Stereo Investigator program to count co-localized cells for more powerful conclusions instead of semi-quantitative quantifications.

Authors thank reviewer for this valuable advice, and we acknowledge that the Stereo Investigator program is a very powerful tool. However, use of this technology is not available to us within a reasonable timeline. In an attempt to get an accurate and representative evaluation, we performed analysis on R0Is that encompassed the entire dentate gyrus in ~11 sections per mouse that were evenly sampled spanning the full length of the structure (one section every 240uM). We believe that this analysis in a sample size of n=10/group, is rigorous enough to draw accurate conclusions. Similar methods have been used and reported by others (e.g. Gobinath et al., 2017 Biology of Sex Differences; Workman et al., 2015 Psychoneuroendocrinology).

10. Figure legends need more detailed explanations of the figures.

We have included greater detail in several figure legends. Additional detail, by figure, is as follows:

Figure 1: (A) “Male and female C57BL/6J mice were placed on control (CON; 10% fat) or high fat (HF; 60% fat) diet at 9 weeks of age, which was continued for the duration of the experiment. After ~3 months on the diet, mice were subjected to glucose tolerance testing (GTT), then injected with EdU (3x) two weeks later. Four weeks after EdU injections, tissue collection was performed.”

Figure 2: (A) “On control diet, females had a greater number of Ki67+ cells in the dorsal hippocampus compared to males, while HF diet reduced the number of Ki67+ cells in the dorsal hippocampus of females only.”

Figure 3: (A) “On control diet, females had a greater number of DCX+ cells in the dorsal hippocampus compared to males, while HF diet reduced the number of DCX+ cells in the dorsal hippocampus of females only.”

Figure 4: (A) “There were no group differences in any area measured. (B) In the dorsal hippocampus, females had a greater number of EdU+/DCX+ cells compared to males, regardless of diet. (C) There were no group differences in any area measured.”

Figure 5: Mice on both control and high fat diet are included in these plots.

11. The extended data present data that are used in correlations presented in the main document. Though not critical, readers may be interested to see the magnitude of the effects, as the diverge between males and females

Authors have added the ‘outer’ microglia data (previously Figure 6-1 in extended data) to Figure 6 so that it is now presented in the main text.
